# A Single CD4 Test with 250 Cells/Mm^3^ Threshold Predicts Viral Suppression in HIV-Infected Adults Failing First-Line Therapy by Clinical Criteria

**DOI:** 10.1371/journal.pone.0057580

**Published:** 2013-02-21

**Authors:** Charles F. Gilks, A. Sarah Walker, Paula Munderi, Cissy Kityo, Andrew Reid, Elly Katabira, Ruth L. Goodall, Heiner Grosskurth, Peter Mugyenyi, James Hakim, Diana M. Gibb, on behalf of the DART Virology Group and Trial Team

**Affiliations:** 1 Department of Medicine, Imperial College, London, United Kingdom; 2 Medical Research Council Clinical Trials Unit, London, United Kingdom; 3 Medical Research Council/ Uganda Virus Research Institute Uganda Research Unit on AIDS, Entebbe, Uganda; 4 Joint Clinical Research Centre, Kampala, Uganda; 5 Clinical Research Centre, University of Zimbabwe, Harare, Zimbabwe; 6 Infectious Diseases Institute, Mulago, Uganda; University of New South Wales, Australia

## Abstract

**Background:**

In low-income countries, viral load (VL) monitoring of antiretroviral therapy (ART) is rarely available in the public sector for HIV-infected adults or children. Using clinical failure alone to identify first-line ART failure and trigger regimen switch may result in unnecessary use of costly second-line therapy. Our objective was to identify CD4 threshold values to confirm clinically-determined ART failure when VL is unavailable.

**Methods:**

3316 HIV-infected Ugandan/Zimbabwean adults were randomised to first-line ART with Clinically-Driven (CDM, CD4s measured but blinded) or routine Laboratory and Clinical Monitoring (LCM, 12-weekly CD4s) in the DART trial. CD4 at switch and ART failure criteria (new/recurrent WHO 4, single/multiple WHO 3 event; LCM: CD4<100 cells/mm^3^) were reviewed in 361 LCM, 314 CDM participants who switched over median 5 years follow-up. Retrospective VLs were available in 368 (55%) participants.

**Results:**

Overall, 265/361 (73%) LCM participants failed with CD4<100 cells/mm^3^; only 7 (2%) switched with CD4≥250 cells/mm^3^, four switches triggered by WHO events. Without CD4 monitoring, 207/314 (66%) CDM participants failed with WHO 4 events, and 77(25%)/30(10%) with single/multiple WHO 3 events. Failure/switching with single WHO 3 events was more likely with CD4≥250 cells/mm^3^ (28/77; 36%) (p = 0.0002). CD4 monitoring reduced switching with viral suppression: 23/187 (12%) LCM versus 49/181 (27%) CDM had VL<400 copies/ml at failure/switch (p<0.0001). Amongst CDM participants with CD4<250 cells/mm^3^ only 11/133 (8%) had VL<400copies/ml, compared with 38/48 (79%) with CD4≥250 cells/mm^3^ (p<0.0001).

**Conclusion:**

Multiple, but not single, WHO 3 events predicted first-line ART failure. A CD4 threshold ‘tiebreaker’ of ≥250 cells/mm^3^ for clinically-monitored patients failing first-line could identify ∼80% with VL<400 copies/ml, who are unlikely to benefit from second-line. Targeting CD4s to single WHO stage 3 ‘clinical failures’ would particularly avoid premature, costly switch to second-line ART.

## Introduction

Most HIV-infected individuals on antiretroviral therapy (ART) in low/middle-income countries are treated following the WHO public health approach [1]: the public sector provides one standard first-line regimen, with alternative drug substitutions for anti-tuberculosis co-therapy/toxicity; when first-line failure occurs, the patient switches to a standard boosted-protease inhibitor (bPI)-based second-line regimen. Current WHO guidelines[2] define failure by virological (>5,000 copies/ml), immunological (CD4 below pre-therapy baseline; 50% fall from on-treatment peak; persistently <100 cells/mm^3^) or clinical criteria after 6 months on ART. Low-income countries differ in their ability to provide laboratory tests to identify first-line failure and support routine follow-up; if available at all, CD4 testing is most common with viral loads (VL) sometimes used to confirm clinical/immunological failure[2]. Routine virological monitoring is rarely available or feasible[3]. Such approaches differ markedly from individualised management in high-income countries, where routine VL monitoring is used to modify initial or subsequent therapy and many drugs are available.

WHO 2010 definition of clinical failure includes WHO 4 and certain WHO stage 3 conditions. Without VLs, it is strongly recommended that immunological criteria confirm clinical failure (noting moderate quality of evidence), but no CD4 threshold value is proposed. We therefore evaluated switches from first to second-line ART in the DART trial[4], [5], particularly considering the unique group randomised to clinically-driven monitoring (CDM) and managed without CD4 counts, but for whom CD4s (and some VLs) were available for retrospective analysis. The aims were to investigate the characteristics of immunological/clinical failures determined without routine VL monitoring and with or without routine real-time CD4 monitoring; and to identify optimal CD4 thresholds to confirm clinical failure and switch to second-line ART when VLs are unavailable.

## Materials and Methods

### Ethics statement

Informed written consent was obtained from each participant and the trial was approved by ethics committees in Uganda, Zimbabwe and the UK.

DART was a randomised controlled trial comparing routine Laboratory (CD4 and toxicity) plus Clinical Monitoring (LCM) with CDM in 3316 symptomatic (WHO stage 2/3/4) HIV-infected ART-naïve adults initiating combination ART with CD4<200 cells/mm^3^ in Uganda/Zimbabwe (ISCRTN13968779)[4]. Participants were enrolled January 2003-October 2004 and initiated co-formulated zidovudine/lamivudine with either tenofovir, abacavir or nevirapine[6].

All participants were reviewed 4-weekly by a nurse, and saw a doctor and had routine lymphocyte subsets (CD4, CD8) at screening, weeks 4 and 12, then 12-weekly. All results from LCM participants were returned to clinicians, whereas for CDM participants CD4 counts were measured but never returned. For all participants, if clinically indicated, diagnostic and other tests could be requested (excluding CD4/total lymphocytes in CDM) and concomitant medications prescribed. VLs were not performed in real-time, but were measured retrospectively on stored plasma samples using Roche Amplicor v1.5.

Participants could substitute alternative antiretrovirals from the nucleoside reverse transcriptase inhibitor (NRTI)/non-NRTI classes in first-line regimens for toxicity, TB-treatment or other reasons; these substitutions did not count as first-line failures. The decision to switch to a second-line bPI-containing regimen for first-line failure was based on clinical criteria in both groups (new/recurrent WHO 4 event (per-protocol); or single or multiple WHO 3 events at clinician discretion), or confirmed CD4<100 cells/mm^3^ on ART (<50 cells/mm^3^ before July 2006) for LCM. Switching was discouraged before 48 weeks on first-line. Clinician's decision to switch also took account of adherence and social circumstances, following standard clinical practice. VL at switch was assayed in all participants enrolling in nested second-line studies from 2007 onwards[7], [8], plus a random sample of other second-line switches (including switches during 2004–2006; 85% assayed on day of switch, 93% within 4 weeks, all within 4 months previously). 336/459 (73%) participants switching after 1 January 2007 chose to join one or both studies. As per the DART protocol, all reported WHO 4 events (but not WHO 3 events) were reviewed against pre-specified criteria by an independent Endpoint Review Committee(ERC) blinded to randomised allocation. This was done retrospectively and did not impact clinical decision-making.

### Statistical analysis

Switch from first- to second-line ART for clinical or immunological failure (the latter only in LCM participants being managed using routine CD4 counts) was the primary outcome of interest. Participants were followed under CDM/LCM strategies until 31 December 2008; this analysis includes only switches to this timepoint and is an exploratory analysis of trial data not specified in the original trial protocol. We used Kaplan-Meier methods to evaluate the time delay from first meeting WHO 4 criteria for switch (in all patients with WHO 4 events after 44 weeks on ART, see below), and actual change in regimen. Although the protocol discouraged switching before 48 weeks, we chose 44 weeks as the cut-off for this analysis to include a small number of patients who switched just before 48 weeks because they returned early for their 48 week visit. The secondary outcome was mortality following switch to second-line. Main exposures considered in those who switched were reported reason for switching, CD4 and VL at switch. Analyses of VL included all available VL which had been measured retrospectively on a subset of participants (see above). Where LCM participants met both immunological (CD4<100 cells/mm^3^) and clinical failure criteria, they were counted as immunological failures. Where CDM participants had both WHO 4 and WHO 3 events, they were counted as WHO 4 failures. Categorical variables were compared using chi-squared/exact tests, continuous variables using t-tests/rank-sum tests. To inform clinical practice when ‘tiebreaker’ VL tests are not available to confirm that clinical/immunological failure has occurred with detectable VL[2], we used receiver-operating-characteristic (ROC) curves to identify the most sensitive and specific (equal weighting) CD4 threshold cut-off for detecting suppressed VL at the point of clinical/immunological first-line failure.

## Results

### Patients monitored clinically without CD4 (or VL) results

1660 CDM participants initiated ART with median(IQR) CD4 86(31–139) cells/mm^3^ and were clinically monitored without CD4 counts for median 5 years. 314(19%) switched to bPI-containing second-line for first-line failure after median(IQR) 3.4(2.5–4.2) years on first-line (only 2 before 48 weeks when switching was discouraged, both at 46 weeks). In those who switched, median(IQR) pre-ART CD4 was 47(14–104) cells/mm^3^ and age at switch 39(34–44) years; 193(61%) were female.

223(13%) CDM participants had new/recurrent WHO 4 events accepted by the ERC after 44 weeks on ART: 187/223 (84%) switched to second-line, 14(6%) died on first-line before switching and 22(10%) had not switched before trial closure. The Kaplan-Meier median(IQR) time to switch after meeting failure criteria was 7(1–23) weeks. The most commonly reported reasons for delaying switch for >8 weeks/not switching were that the WHO 4 event was judged unrelated to ART failure by the clinician (45%) or because of drug-drug interactions between rifampicin and bPI (32%) (Table 1; n = 44). An additional 20 patients switched to second-line for WHO 4 events eventually judged not to meet pre-defined protocol criteria by the ERC, leading to a total 207/314 (66%) switches in CDM being for WHO 4 events.

**Table 1:** Reasons for not switching when first met immunological/clinical criteria for switch in patients receiving and not receiving CD4 monitoring.

	CD4 monitoring (LCM)	No CD4 monitoring (CDM)
Total met clinical (WHO 4 event) or immunological (confirmed CD4<100 cells/mm^3^) criteria for switch and either switched >8 weeks later or did not switch	132 (100%)	
Total met clinical criteria (WHO 4 event) for switch and either switched >8 weeks later or did not switch		100 (100%)
Reason for not initially switching patient reported (details below)*	42 (32%)	44 (44%)
No reason reported but switched within 6 months	56 (42%)	33 (33%)
Reason not reported, not switched within 6 months	34 (26%)	23 (23%)
* Switched after 6 months*	*27*	*21*
* Died or last seen alive and not switched*	*7*	*2*
Reason for not switching when first met criteria (% of those reporting a reason)	42 (100%)	44 (100%)
Patient judged to be doing well	30 (71%)	20 (45%)
* CD4>200*	*19*	*N/A*
* CD4 100-200 or <100 but still increasing*	*9*	*N/A*
* only been on ART for ∼1 year*	*0*	*3*
* clinical judgement that event not related to ART failure* **	*2*	*12*
* client felt well and did not want to switch*	*0*	*5*
On TB treatment with rifampicin	10 (24%)	14 (32%)
Poor adherer/defaulter	1 (2%)	4 (9%)
Too ill to switch	0 (0%)	4 (9%)
Oversight: should have switched	1 (2%)	2 (5%)

* based on a retrospective request for reasons why patients had not switched within 8 weeks of first meeting protocol switch criteria.

** eg only presumptive diagnosis, responded to treatment for the clinical event, event judged related to recent period off ART, patient being monitored and doing well.

In the same period, 70 multiple (within 60 days) and 392 single WHO 3 events were reported in participants not switching for WHO 4 events. Clinicians used clinical judgement to assess which of these events were likely first-line ART failure, leading to 30(10%) and 77(25%) of the 314 CDM switches being for multiple or single WHO 3 events respectively (Table 2). More switches for multiple/single WHO 3 events occurred over calendar time (2004–2008) reflecting wider promotion of WHO 3 events as switch criteria in WHO 2006 guidelines[9]; eg 85% of pre-2007 switches were due to WHO 4 events compared to 53% subsequently.

**Table 2:** CD4 and VL at switch to second-line for first-line failure in patients not receiving CD4 or VL count monitoring (CDM).

Clinical failure criteria triggering switch	Number switching (%)	Median (IQR) CD4 at switch	n≥250 cells/mm^3^ at switch (%)	n<50 cells/mm^3^ at switch (%)	n died within 1 year of switch (%)	VL assayed‡ (% of total)	n with VL<400 copies/ml (% of those with VL assayed)	Median VL in those >400 copies/ml	ROC area under the curve for CD4 predicting VL<400 copies/ml
**Total switched**	**314 (100%)**	**56 (15–196)**	**64 (20%)**	**151 (48%)**	**44 (14%)**	**181 (58%)**	**49 (27%)**	**81,565**	0.91**
WHO 4	207† (66%)	47 (15–165)	33 (16%)	105 (51%)	39 (19%)	103 (50%)	27 (26%)	89,907	0.92
Multiple WHO 3	30 (10%)	19 (9–79)	3 (10%)	18 (60%)	1 (3%)	20 (67%)	3 (15%)	36,125	0.86
Single WHO3	77 (25%)	102 (23–364)	28 (36%)	28 (36%)	4 (5%)	58 (75%)	19 (33%)	80,837	0.90
		p = 0.0006	p = 0.0002	p = 0.04	p = 0.003	p<0.0001*	p = 0.29	p = 0.59	

* p = 0.84 adjusted for whether whether enrolled in second-line studies (p<0.0001) and whether switched to second-line before or after 1 Jan 2007 (p = 0.15), sex (p = 0.45) and age at switch (p = 0.54). More participants switched for non-WHO 4 reasons later in the trial reflecting wider promotion of WHO 3 events as switch criteria in WHO 2006 guidelines[9]; see Results.

** ROC area under the curve  =  0.90 (95% CI 0.83–0.98) in 117 patients enrolled in second-line studies, vs 0.93 (95% CI 0.86–0.99) in 64 patients not enrolled in second-line studies.

† 66/207 (32%) also had WHO 3 events in the month prior to switch (of which 37 were oral candida and 19 were weight loss >10%)

‡ retrospectively on stored plasma

Note: ROC  =  receiver operating characteristic, see Figure 2. Two switches 46 weeks after ART initiation: all others ≥52 weeks.

CD4 counts were performed 12-weekly in all CDM participants, but, as not returned, did not influence the decision to switch. Although the median(IQR) CD4 count at switch was 56(15–196), 64(20%) participants had CD4 ≥250 cells/mm^3^ (Table 2). Switching for failure determined by a single WHO 3 event was significantly more likely to occur with CD4≥250 cells/mm^3^ (36%) compared to multiple WHO 3 (10%) or WHO 4 (16%) events (p = 0.0002).

Most WHO 4 events triggering switch had relatively similar proportions with CD4≥250 cells/mm^3^ and <50 cells/mm^3^ (Table 3), with the exception of cryptococcal meningitis, where one third (11 events) triggered switch with CD4≥250 cells/mm^3^. Interestingly CD4 was ≥250 cells/mm^3^ in three of the four switches to second-line triggered by lymphoma. Although generally considered a less severe WHO 4 event, median(IQR) CD4 at switch triggered by oesophageal candidiasis was only 30(8–68) cells/mm^3^. Weight loss, severe bacterial infection (SBI) and diarrhoea were the main single WHO 3 events triggering switch with CD4≥250 cells/mm^3^ (44%, 43% and 100% respectively), likely reflecting their frequency in adults irrespective of HIV status or CD4. However, combinations of ≥2 WHO 3 events triggered switch at lower CD4 counts, similar to WHO 4 events.

**Table 3:** Clinical events triggering switch after 44 weeks on ART in patients monitored without CD4 cell counts (CDM).

	No CD4 monitoring (CDM)				
Clinical events triggering switch after 44 weeks on ART	N[accepted by ERC] (%)	Median (IQR) CD4	n≥250 cells/mm^3^(%)	n<50 cells/mm^3^(%)	n died within 1 year of switch(%)
**WHO 4**	**207 (100%)**	**47 (15–165)**	**33(16%)**	**105(51%)**	**39(19%)**
Oesophageal candidiasis	76 [70] (37%)	30 (8–68)	3(4%)	47(62%)	8(11%)
Cryptococcal meningitis	33 [28] (16%)	70 (16–270)	11(33%)	15(45%)	11(33%)
Extra–pulmonary TB	26 [22] (13%)	78 (23–188)	3 (12%)	9 (35%)	5 (19%)
HIV wasting	23 [17] (11%)	58 (12–149)	4 (17%)	9 (39%)	7 (30%)
Herpes simplex, mucotaneous	14 [10] (7%)	30 (16–242)	3 (21%)	8 (57%)	2 (14%)
Cryptosporidiosis	13 [12] (6%)	30 (18–203)	3 (23%)	8 (62%)	1 (8%)
PCP	7 [3] (3%)	17 (5–72)	0 (0%)	5 (71%)	1 (14%)
Lymphoma	4 [3] (2%)	373 (178–460)	3 (75%)	1 (25%)	3 (75%)
KS	4 [3] (2%)	149 (66–244)	1 (25%)	1 (25%)	1 (25%)
Toxoplasmosis	2 [2] (1%)	28 (5,52)	0 (0%)	1 (50%)	0 (0%)
CMV	2 [2] (1%)	410 (244,575)	1 (50%)	0 (0%)	0 (0%)
**Multiple WHO 3**	**30 (100%)**	**19 (9–79)**	**3 (10%)**	**18 (60%)**	**1 (3%)**
Weight loss, oral candida	12 (40%)	12 (9–57)	1 (8%)	8 (67%)	1 (8%)
Weight loss, SBI	6 (20%)	54 (10–71)	0 (0%)	3 (50%)	0 (0%)
Oral candida, SBI	4 (13%)	26 (14–166)	1 (25%)	3 (75%)	0 (0%)
Oral candida, pulmonary TB	2 (7%)	8 (3,13)	0 (0%)	2 (100%)	0 (0%)
Multiple SBI	2 (7%)	116 (79,153)	0 (0%)	0 (0%)	0 (0%)
**Single WHO3**	**77 (100%)**	**102 (23–364)**	**28 (36%)**	**28 (36%)**	**4 (5%)**
Weight loss	41 (53%)	224 (37–409)	18 (44%)	11 (27%)	1 (2%)
Oral candida	20 (26%)	31 (12–138)	4 (20%)	12 (60%)	0 (0%)
SBI	7 (9%)	55 (19–303)	3 (43%)	3 (43%)	1 (14%)
Pulmonary TB	5 (6%)	71 (27–98)	1 (20%)	2 (40%)	0 (0%)
Diarrhoea	2 (3%)	490 (327,652)	2 (100%)	0 (0%)	1 (50%)

Note: SBI  =  severe bacterial infection; OHL = oral hairy leukoplakia; ERC = Endpoint Review Committee (blinded to randomised group). Data not shown for events with only 1 associated switch: visceral herpes simplex, HIV encephalopathy, recurrent pneumonia, weight loss+persistent fever, weight loss+oral candida+SBI, oral candida+OHL, pulmonary TB+SBI, HIV nephropathy, OHL). Additional new/recurrent WHO events which occurred during the first year on ART are included in the main trial report[4], but not here as switch to second-line for first-line failure only occurred after 1 year.

CDM participants switching with WHO 4 events were more likely to die within a year of switch than those switching with multiple or single WHO 3, consistent with their severity (19% versus 3% versus 5% respectively, exact p = 0.002, Table 2). Interestingly, subsequent mortality was similar in those switching at high and lower CD4 counts: 17% (11/64) CDM participants switching with CD4≥250 cells/mm^3^ died within a year of switch versus 13% (33/250) switching with CD4<250 cells/mm^3^ (p = 0.41, p = 0.51 adjusted for WHO 3/4 events); and 27% (9/33) CDM participants switching for WHO 4 events with CD4≥250 cells/mm^3^ died within 1 year versus 17% (30/174) with CD4≤ 250 cells/mm^3^ (p = 0.22).

**Figure 1:**
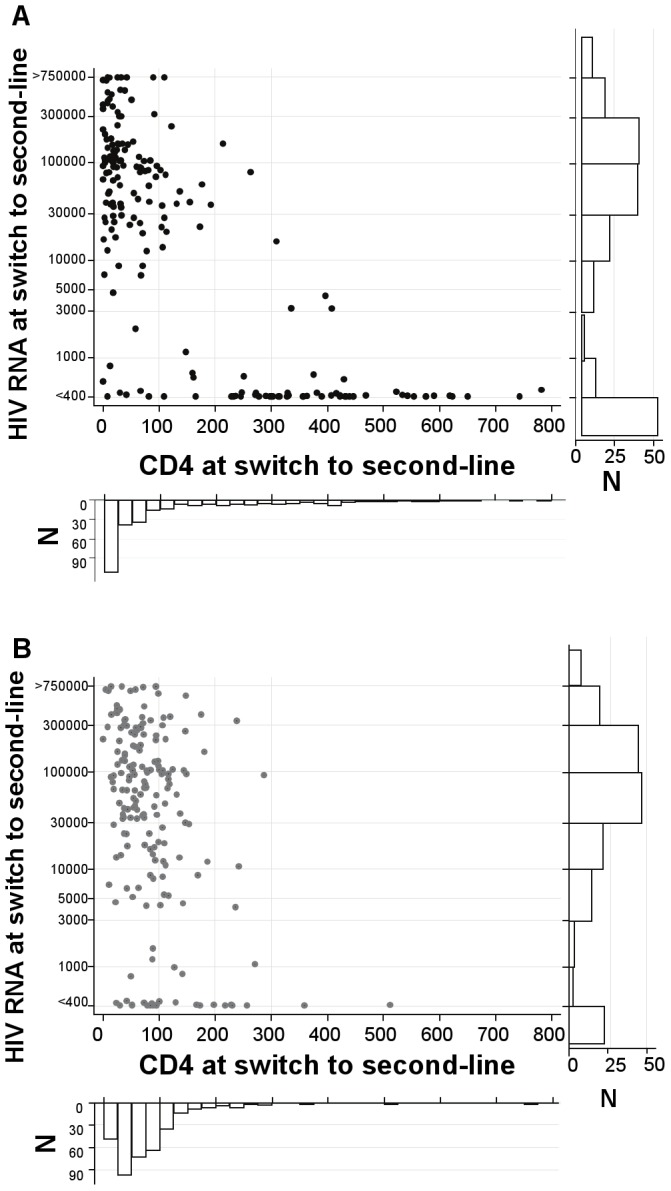
VL and CD4 at switch to second-line for first-line failure in patients receiving and not receiving CD4 count monitoring. (a) in patients not receiving routine CD4 count monitoring (CDM: 20% >250 cells/mm3). (b) in patients receiving routine CD4 count monitoring (LCM: 2% >250 cells/mm3). Footnote 1 Points at >750000 and <400 have a small amount of ‘jitter’ added so that all observations are visible.

Overall 181(58%) CDM participants had VL at switch to second-line assayed retrospectively. 49(27%) had VL<400 copies/ml, with similar proportions across reasons for triggering switch (p = 0.29) (Table 2). Thus 3.7 ‘tie-breaker’ VL tests at clinical failure would be needed to prevent one switch with suppressed VL. There was a very wide range of VLs in clinically-monitored participants failing and switching with CD4<100 cells/mm^3^ (Figure 1(a)). In contrast, most with CD4>250 cells/mm^3^ had suppressed VL.

**Figure 2:**
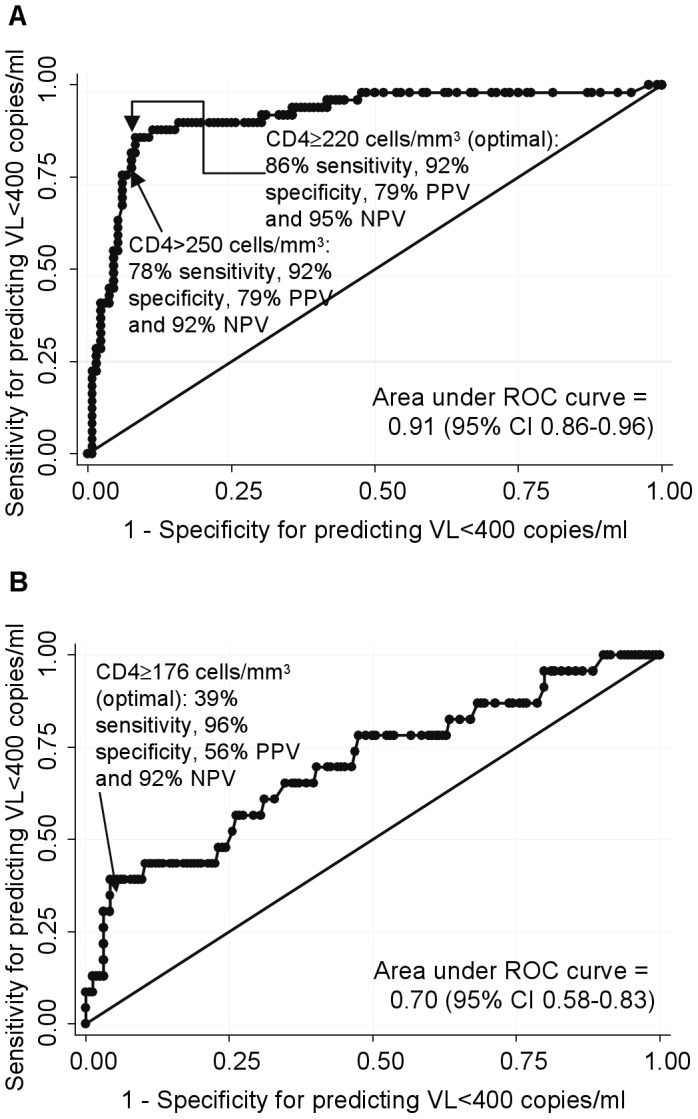
Ability of a single CD4 at switch to second-line for first-line failure to predict VL. (a) in patients not receiving routine CD4 count monitoring (CDM: 20% >250 cells/mm3). (b) in patients receiving routine CD4 count monitoring (LCM: 2% >250 cells/mm3). Footnote 2 Receiver operator curves (ROC) show how the sensitivity and specificity of CD4 thresholds for predicting VL<400 copies/ml varies as CD4 increases from 1 to 788 (CDM) or 505 (LCM) cells/mm^3^. The straight line indicates performance no better than chance. The threshold with the greatest probability of correctly classifying each CD4 count according to whether it has VL<400 copies/ml or not is indicated with sensitivity (proportion with VL<400 c/ml who have CD4≥threshold), specificity (proportion with VL≥400 c/ml who have CD4 <threshold), positive predictive value (proportion of patients with CD4 ≥threshold who have VL<400 c/ml) and negative predictive value (proportion of patients with CD4 <threshold who have VL≥400 c/ml).

To inform practice where VL testing is unavailable or performed off-site (when return of results may be delayed considerably), we evaluated the predictive ability of a single tie-breaker CD4 count at clinically-triggered switch to identify participants with VL<400 copies/ml, using data on VLs assayed retrospectively on stored samples and CD4 counts which had not been returned to clinicians. VL was <400 copies/ml in 38/48(79%) with CD4≥250 cells/mm^3^ versus only 11/133(8%) with CD4<250 cells/mm^3^ (p<0.0001). The area under the receiver-operating-characteristic (ROC) curve (Figure 2(a)) was 0.91 (95% CI 0.86–0.96), with an optimal threshold where most observations were correctly classified (90%) of CD4≥220 cells/mm^3^. This cutoff had 86% sensitivity, 92% specificity, 79% positive predictive value and 95% negative predictive value for identifying participants with VL<400 copies/ml (LR+ = 10.3, LR- = 0.16). ROC areas were similar according to reason for switch and in those joining and not joining second-line studies (Table 2). Therefore a threshold of >250 cells/mm^3^, a close but more likely cut-off for a CD4 point-of-care assay, would capture most individuals without virological failure, for whom switching could be premature and unnecessary.

### Patients monitored clinically with 12-weekly CD4 but no VL results

1656 LCM participants initiated ART with median(IQR) CD4 86(32–140) cells/mm^3^ and were clinically monitored together with 12-weekly CD4 counts for median 5 years. 361(22%) switched to bPI-containing second-line for first-line failure after a median(IQR) 2.8(2.1–3.8) years on first-line (shorter than in CDM, p = 0.0001) (only 1 <48 weeks). In those who switched, median(IQR) pre-ART CD4 was 42(17–85) cells/mm^3^ and age at switch 39(34–45) years; 201(57%) were female.

326(20%) had a new/recurrent WHO 4 event accepted by the ERC or met immunological failure criteria after the first 44 weeks on ART. 286/326 (88%) switched to second-line, 6(2%) died on first-line before switching and 34(10%) had not switched before trial closure. The Kaplan-Meier median(IQR) time to switch after meeting the criteria was 4(2–20) weeks, similar to CDM (p = 0.70). The most commonly reported reasons for delaying switch for >8 weeks or not switching was high CD4s (71%) or because of drug-drug interactions between rifampicin and bPI (24%) (Table 1; n = 42). An additional 75 participants switched to second-line ART for WHO 4 events not judged to meet pre-defined protocol criteria by the ERC, single/multiple WHO 3 events or other CD4-related reasons.

As expected, since CD4 decline generally precedes development of WHO 4 events, most (73%, 265/361) LCM participants who switched failed by the CD4<100 cells/mm^3^ protocol criteria. An additional 37(10%) switched for WHO 4 events; 43(12%) for other CD4 reasons (mainly rapid CD4 decline to >100 cells/mm^3^); and only 6(2%) and 10(3%) with multiple or single WHO 3 events respectively (Table 4). Over time, more switches occurred for CD4s which were low but ≥100 cells/mm^3^; for example, 2% pre-2007 switches were due to CD4 ≥100 cells/mm^3^ compared to 24% subsequently, reflecting changes in WHO2006 guidelines[8].

**Table 4:** CD4 and VL at switch to second-line for first-line failure in patients receiving 12-weekly CD4 count monitoring, but no VL monitoring (LCM).

Clinical or immunological failure criteria triggering switch	Number switching (%)	Median (IQR) CD4at switch	n≥250 cells/mm^3^ at switch (%)	n<50 cells/mm^3^ at switch (%)	n died within 1 year of switch (%)	VL assayed‡ (% of total)	n with VL<400 copies/ml (% of those with VL assayed)	Median VL in those >400 copies/ml	ROC area under the curve for CD4 predicting VL<400 copies/ml
Total switched	361 (100%)	63 (36–95)	7 (2%)	145 (40%)	23 (6%)	187 (52%)	23 (12%)	86,568	0.70‡‡
CD4<100 cells/mm^3^	265† (73%)	47 (30–71)	0	140 (53%)	8 (3%)	122 (46%)	9 (7%)	99,784	0.64
Other CD4*	43 (12%)	113 (92–153)	3 (7%)	1** (2%)	2 (5%)	29 (67%)	6 (21%)	33,340	0.67
WHO 4	37 (10%)	116 (83–179)	3 (8%)	4 (11%)	12 (32%)	23 (62%)	6 (26%)	87,536	0.64
Multiple WHO 3	6 (2%)	130 (95–241)	0	0	1 (17%)	5 (83%)	0 (0%)	59,990	N/A
Single WHO3	10 (3%)	118 (103–165)	1 (10%)	0	0 (0%)	8 (80%)	2 (25%)	166,858	1.00
		p = 0.0001	p<0.0001	p<0.0001	p<0.0001	p = 0.005††	p = 0.03	p = 0.08	

* mostly CD4 declines to just over 100 cells/mm^3^

† 65/265 (25%) also had WHO 4 events at the time of switch (plus 1 of the 43 switching for other CD4 reasons)

** CD4 <50 cells/mm^3^ on day of switch: did not have any previous CD4 <50 cells/mm^3^ or 2 previous CD4s <100 cells/mm^3^

‡ retrospectively on stored plasma

†† p = 0.95 in a multivariable logistic regression model for VL assayed (yes/no) adjusted for whether switched to second-line before or after 1 Jan 2007 (p = 0.12), whether or not joined second-line studies (p = 0.05), sex (p = 0.89) and age at switch (p = 0.61). More patients switched for other CD4 reasons later in the trial, reflecting wider promotion of other immunological criteria as switch criteria in WHO 2006 guidelines[9]; see Results.

‡‡ ROC area under the curve  =  0.62 (95% CI 0.45–0.79) in 115 patients enrolled in second-line studies, vs 0.78 (95% CI 0.58–0.99) in 72 patients not enrolled in second-line studies.

Note: ROC  =  receiver operating characteristic, see Figure 2. One switch 34 weeks after ART initiation: all others ≥48 weeks.

The median(IQR) CD4 count at switch was 63(36–95) cells/mm^3^. In contrast to CDM participants, but reflecting clinician reluctance to switch LCM participants with WHO events and high CD4s (Table 1), only 7(2%) CD4-monitored participants switched with CD4≥250 cells/mm^3^ (3 for WHO 4; 1 single WHO 3; 3 other CD4 reasons, all in participants with substantial prior CD4 variability). Mortality following switch was highest in participants who switched for WHO 4 events before meeting immunological failure criteria (32% (12/37); p<0.0001)

Overall 187(52%) participants had VL at second-line switch assayed retrospectively. 23(12%) had VL<400 copies/ml (Table 4; p<0.0001 versus CDM). Thus 8.1 ‘tie-breaker’ VL tests at immunological/clinical failure would be needed to prevent one switch with suppressed VL. Fewer participants switching for CD4<100 cells/mm^3^ had VL<400 copies/ml (7%) than those meeting other CD4 or clinical criteria (p = 0.03). Those with CD4<100 cells/mm^3^ had a very wide range of VLs (Figure 1(b)), similarly to participants monitored without CD4s.

In those receiving 12-weekly CD4 counts, CD4 at switch was a much poorer predictor of suppressed VL (Figure 2(b)); with area under the LCM ROC curve 0.70 (95% CI 0.58–0.73) (similar according to reason for switch and participation in second-line studies). This may be because only 14(4%) had CD4 above the optimal threshold of 220 cells/mm^3^ for identifying VL suppression found in CDM, since those LCM participants with high CD4, in whom VL was more likely suppressed, were rarely switched by clinicians (Table 1). In fact, 54% (13/24) of LCM participants who switched with VL<400 copies/ml actually had CD4<110 cells/mm^3^ (Figure 1(b)) (median(range) 3.4(1.1–4.9) years on first-line), highlighting that completely accurate CD4-based prediction of suppressed VL is impossible.

Interestingly, irrespective of monitoring strategy, virological-responders/immunological-non-responders with VL<400 copies/ml but CD4<110 cells/mm^3^ had excellent response to bPI-containing second-line ART. In 14 participants (10LCM,4CDM) the median(IQR) CD4 increase 24 weeks after switching was +129 (+42,+216) cells/mm^3^ from median(IQR) 70(38–94) cells/mm^3^ at switch (4 others (3LCM,1CDM) died before 24 weeks on second-line).

## Discussion

Most public sector ART clinics in low-income settings have very limited laboratory capacity to monitor patients on therapy, so justifying and prioritising services provided to support clinical monitoring is critical. DART has already shown that routine CD4 monitoring alone from the second year of ART has a small but important impact on survival[4]. WHO recommends the use of VLs to confirm immunological/clinical failure[2]. Currently, neither are widely accessible; eg in April-June 2011, there were only 50 functional CD4 machines in 449 ART clinics in Malawi[10] and in early 2012, only 4/59 ART centres in Malawi, Zimbabwe, Uganda, including hospitals, had the possibility to monitor VL, including off-site[3]; even if theoretically functional, lack of electricity/consumables/personnel may further reduce their availability in practice. Further analysis of the unique group of DART participants failing first-line ART who were clinically-monitored without routine CD4/VL tests, but with retrospective CD4 and VLs available, now shows the utility of multiple, but not single, WHO 3 events as clinical failure criteria in the absence of any CD4 monitoring; and that, where access to single CD4 tests is available, a CD4 tiebreaker at a 250 cells/mm^3^ threshold could identify ∼80% of those failing clinically with VL<400 copies/ml who may be unlikely to benefit from switching to more costly second-line therapy.

Despite WHO recommendations to use a VL ‘tie-breaker’ test to confirm clinical/immunological failure[2], access to expensive HIV RNA testing is unlikely to improve soon given the current financial crisis. Point-of-care VL testing could dramatically change this, but is unlikely to become available for several years, and may still be relatively costly. Meanwhile, many public sector ART programmes will continue to monitor ART patients with negligible access to VL. In contrast, CD4 testing is more widely accessible[3], and point-of-care devices already in evaluation will soon increase coverage[11]. However, the sheer volume of testing will remain challenging, as evidenced by stockouts even of simple HIV tests[3] and given the 6.6 million adults/children receiving ART in low/middle-income settings[12]. Making routine CD4 monitoring available to all would require significant additional investments in laboratory infrastructure, personnel and consumables, which may not be possible given the current financial situation, particularly as, at current costs, it is not cost-effective for most African countries[13], [14], and a more pressing priority is to rollout ART to more who need it. Additional benefits of routine VL over CD4 monitoring are small[15], [16] or negligible[17] and even less cost-effective[18]. It is therefore essential to consider parsimonious ways to use CD4 testing without VLs to support clinical monitoring in the critical decision of when to switch to second-line.

As our analysis investigated characteristics of those patients switched to second-line, we did not (and cannot) estimate the overall accuracy of CD4 (or clinical) criteria for identifying virological failure in all individuals on treatment. However, our data clearly confirm that monitoring for clinical failure alone over-identifies immunological failure, potentially resulting in unnecessary and premature switching to more costly second-line ART[14], [19]: 20% of clinical failures/switches had CD4>250 cells/mm^3^. The low CD4 nadir in DART participants (10% had pre-ART CD4 <10 cells/mm^3^) may have contributed to this, with patients at long-term risk for events such as lymphoma, despite immune reconstitution. Furthermore, 12% of CD4-monitored and 27% of clinically-monitored participants had suppressed VL at failure/switch, as previously reported[19]-[22]. Discordance between clinical, immunological and virological failure at any single timepoint is expected, as they track different processes. Nor is failure always absolute: eg 50% of patients with virological failure and genotypic NNRTI resistance in one South African cohort re-suppressed while receiving an NNRTI[23]. In resource-limited settings with access to single confirmatory laboratory tests, the challenge of how to deal practically with discordance remains.

Whilst relatively short periods on ART leading to incomplete immune reconstitution may account for some discrepancies in previous studies[19], [20], the 18 participants switching with CD4<110 cells/mm^3^ and VL<400 copies/ml in DART had been on first-line ART for median 3.4 years. Some had variable CD4 responses on first-line, with periods of low CD4 without documented non-adherence; others had never responded immunologically, similarly to Kantor *et al* where 3/7 patients with persistent CD4<100 cells/mm^3^ had undetectable VL[20]. Most DART participants with VL<400 copies/ml but very low CD4 at failure benefited considerably immunologically from second-line switch; however 22% (4/18) died shortly after switching. Given increased mortality risks at CD4<100 cells/mm^3^ even with suppressed VL in resource-rich[24], [25] and resource-limited settings[26], there may be clinical benefits from switching this specific group of discordant responders with very low CD4 from an NRTI/NNRTI-based first-line to a bPI-based second-line regimen. Of interest, we also observed relatively high mortality in participants switching with high CD4 counts and WHO 4 events, possibly reflecting that developing such events despite apparently high absolute CD4 counts may indicate underlying functional immune deficits that may themselves impact mortality risk.

Those ‘failing ART’ clinically with only single WHO 3 events can be viewed in two ways. The fact that nearly 40% had CD4>250 cells/mm^3^ and 33% VL<400 copies/ml demonstrates lack of sensitivity of these events for ART failure, probably because of their frequency in the underlying population irrespective of HIV status. Whilst our results suggest single WHO 3 events should not trigger switch in isolation, conversely 64% of this group had CD4≤250 cells/mm^3^ and 67% VL>400 copies/ml, greater than the wider population on first-line. Targeting them for confirmatory tiebreak CD4 testing could therefore identify additional ART failures whilst avoiding premature switching. Of note, participants switching with multiple WHO 3 events had lower CD4 and higher VL at switch than single WHO 3 events. Adding WHO 3 (single or multiple) events to the WHO 4 criteria for clinical failure increased the numbers identified by about 50%.

One potential limitation of our study is that clinical monitoring was conducted by nurses and clinicians in relatively well-supported, staffed and supervised sites with no ART stock-outs. DART had excellent retention (just 7% loss to follow-up over five years) and 5-year survival (87% CDM, 90% LCM). Among participants randomised to CDM, CD4s were measured but not returned and health-workers remained blinded to CD4s throughout the trial. This enabled us to perform analyses impossible outside a trial design such as DART. Whilst several WHO 4 events triggering switch in DART were not considered to meet criteria for trial endpoints on independent review, we included them in these analyses. In ART programmes, more WHO 3/4 events might be over-diagnosed (with clinicians conservatively ascribing clinical episodes as WHO 3/4 events), but these would likely occur with high CD4 and VL<400 copies/ml supporting generalisability of our findings. DART participants were severely immune-compromised (median 86 cells/mm^3^) when initiating ART: generalizability to programmes initiating ART earlier is unknown. However, such patients would take longer to fail on first-line therapy, and therefore it is plausible that a greater (rather than smaller) proportion of clinical events on first-line would be single WHO 3 rather than multiple WHO 3 or 4 events, in whom we found greatest VL suppression. Our findings may therefore be more, rather than less generalizable, to such settings.

The DART protocol only included one of the three WHO immunological failure criteria (confirmed <100 cells/mm^3^) for LCM participants (although a small number of participants switched for other CD4 concerns before this). The other criteria require a series of CD4s (50% decline from peak) or a pre-ART CD4 (drop below pre-ART baseline) and were not included in the protocol because they were judged impractical in settings with limited access to CD4 testing and are anyway not validated; it was considered that switching should be determined only by known predictors of mortality on ART (ie overall CD4, not declines or drops below baseline). These other immunological failure criteria also generally lead to switch at higher CD4 counts and might be expected to be associated with higher rates of VL suppression at switch than demonstrated here, but we cannot assess this. In addition, as real-time VL monitoring was not performed, we cannot evaluate the VL>5000 copies/ml WHO criteria for switching without immunological or clinical failure[2], nor investigate the performance of CD4 criteria in identifying this threshold value. Figure 1 shows that most participants switching for immunological/clinical failure with VL>400 copies/ml had values around or above this threshold.

The unique opportunity afforded by the DART trial design to evaluate the value of using CD4 testing parsimoniously to support clinical monitoring when VL monitoring is unavailable, provides clear evidence that a single CD4 tie-breaker at clinical failure with a 250 cells/mm^3^ threshold can identify patients who have suppressed VL, providing the potential to reduce unnecessary switching to costly second-line ART. Adding a single CD4 count in those failing clinically could therefore improve the specificity (and positive predictive value) of clinically identified failure for virological failure: clearly a single count cannot improve the sensitivity for detecting virological failure in patients without clinical events. Our results further suggests that multiple (but not single) WHO 3 events should be considered as well as WHO 4 events in the definition of clinical failure when CD4 testing is unavailable. If limited CD4 testing is possible, targeting this to patients with a single WHO stage 3 event could identify additional failures with detectable VL whilst still avoiding premature, costly switching to second-line. Programmes in low-income countries that are considering how to scale-up laboratory services to rationally support regular clinical follow-up can use these results to plan how to widen access to CD4 monitoring taking advantage of new point-of-care technologies.

## References

[1] Gilks CF, Crowley S, Ekpini R, Gove S, Perriens J, et al (2006) The WHO public-health approach to antiretroviral treatment against HIV in resource-limited settings. Lancet 368: 505–510. 16890837 10.1016/S0140-6736(06)69158-7

[2] World Health Organisation (2010) Antiretroviral therapy for HIV infection in adults and adolescents: Recommendations for a public health approach. 2010 revision. WHO Geneva 23741771

[3] Abongomera G, Namata H, Nkhata M, Muzambi M, Seeley J, et al.. (2012) Lablite: baseline mapping survey of decentralised ART service provision in Malawi, Uganda and Zimbabwe. XIX International AIDS Conference. Washington DC, USA, 22–27 July.Abstract LBPE57.

[4] DART Trial Team (2010) Routine versus clinically driven laboratory monitoring of HIV antiretroviral therapy in Africa (DART): a randomised non-inferiority trial. Lancet 375: 123–131. 20004464 10.1016/S0140-6736(09)62067-5 PMC2805723

[5] DART Trial Team (2008) Fixed duration interruptions are inferior to continuous treatment in African adults starting therapy with CD4 cell counts < 200 cells/microl. Aids 22: 237–247. 18097226 10.1097/QAD.0b013e3282f2d760

[6] DART Trial Team (2008) Twenty-four-week safety and tolerability of nevirapine vs. abacavir in combination with zidovudine/lamivudine as first-line antiretroviral therapy: a randomized double-blind trial (NORA). Trop Med Int Health 13: 6–16. 10.1111/j.1365-3156.2007.01973.x PMC2635484 18290996

[7] Gilks CF, Walker AS, Dunn DT, Gibb DM, Ben Kikaire B, et al.. (in press) Lopinavir/ritonavir monotherapy after 24 weeks of second-line anti-retroviral therapy in Africa: a randomised controlled trial (SARA) Antivir Ther. 10.3851/IMP2253 22814125

[8] Mambule I, Walker AS, Reid A, Ssali F, Munderi P, et al.. (2011) Second-line boosted protease-containing therapy: assessing the impact of maintaining 3TC vs switching to ddI in addition to 2 drugs from new classes in a randomized comparison. 18th Conference on Retroviruses and Opportunistic Infections.Boston, 27 Feb-2 Mar (Abstract 541).

[9] World Health Organisation (2006) Antiretroviral therapy for HIV infection in adults and adolescents in resource-limited settings: towards universal access. Recommendations for a public health approach. WHO Geneva

[10] Government of Malawi Ministry of Health (2011) Quarterly HIV Programme Report.

[11] Jani IV, Sitoe NE, Alfai ER, Chongo PL, Quevedo JI, et al (2011) Effect of point-of-care CD4 cell count tests on retention of patients and rates of antiretroviral therapy initiation in primary health clinics: an observational cohort study. Lancet 10.1016/S0140-6736(11)61052-0 21951656

[12] World Health Organisation (2011) HIV treatment reaching 6.6 million people, but majority still in need. 22026289

[13] Medina Lara A, Kigozi J, Amurwon J, Muchabaiwa L, Nyanzi Wakholi B, et al (in press) Cost effectiveness analysis of clinically driven versus routine laboratory monitoring of antiretroviral therapy in Uganda and Zimbabwe. PlosONE 10.1371/journal.pone.0033672 PMC3335836 22545079

[14] Walker AS, Gibb DM (2011) Monitoring of highly active antiretroviral therapy in HIV infection. Curr Opin Infect Dis 24: 27–33. 21150591 10.1097/QCO.0b013e3283423e0e

[15] Laurent C, Kouanfack C, Laborde-Balen G, Aghokeng AF, Mbougua JB, et al (2011) Monitoring of HIV viral loads, CD4 cell counts, and clinical assessments versus clinical monitoring alone for antiretroviral therapy in rural district hospitals in Cameroon (Stratall ANRS 12110/ESTHER): a randomised non-inferiority trial. Lancet Infect Dis 11: 825–833. 21831714 10.1016/S1473-3099(11)70168-2

[16] Mermin J, Ekwaru JP, Were W, Degerman R, Bunnell R, et al (2011) Utility of routine viral load, CD4 cell count, and clinical monitoring among adults with HIV receiving antiretroviral therapy in Uganda: randomised trial. BMJ 343: d6792. 22074711 10.1136/bmj.d6792 PMC3213241

[17] Jourdain G, Ngo-Giang-Huong N, S Le Coeur S, Traisaithit P, Barbier S, et al.. (2011) PHPT-3: a randomized clinical trial comparing CD4 vs viral load ART monitoring/switching strategies in Thailand. 18th Conference on Retroviruses and Opportunistic Infections. Boston, 27 Feb-2 Mar (Abstract 44).

[18] Kahn JG, Marseille E, Moore D, Bunnell R, Were W, et al (2011) CD4 cell count and viral load monitoring in patients undergoing antiretroviral therapy in Uganda: cost effectiveness study. BMJ 343: d6884. 22074713 10.1136/bmj.d6884 PMC3213243

[19] van Oosterhout JJ, Brown L, Weigel R, Kumwenda JJ, Mzinganjira D, et al (2009) Diagnosis of antiretroviral therapy failure in Malawi: poor performance of clinical and immunological WHO criteria. Trop Med Int Health 14: 856–861. 19552661 10.1111/j.1365-3156.2009.02309.x

[20] Kantor R, Diero L, Delong A, Kamle L, Muyonga S, et al (2009) Misclassification of first-line antiretroviral treatment failure based on immunological monitoring of HIV infection in resource-limited settings. Clin Infect Dis 49: 454–462. 19569972 10.1086/600396 PMC4859427

[21] Moore DM, Awor A, Downing R, Kaplan J, Montaner JS, et al (2008) CD4+ T-cell count monitoring does not accurately identify HIV-infected adults with virologic failure receiving antiretroviral therapy. J Acquir Immune Defic Syndr 49: 477–484. 18989232 10.1097/QAI.0b013e318186eb18

[22] Reynolds SJ, Nakigozi G, Newell K, Ndyanabo A, Galiwongo R, et al (2009) Failure of immunologic criteria to appropriately identify antiretroviral treatment failure in Uganda. AIDS 23: 697–700. 19209067 10.1097/QAD.0b013e3283262a78 PMC2720562

[23] Hoffmann CJ, Charalambous S, Sim J, Ledwaba J, Schwikkard G, et al (2009) Viremia, resuppression, and time to resistance in human immunodeficiency virus (HIV) subtype C during first-line antiretroviral therapy in South Africa. Clin Infect Dis 49: 1928–1935. 19911963 10.1086/648444 PMC2789416

[24] Mocroft A, Ledergerber B, Zilmer K, Kirk O, Hirschel B, et al (2007) Short-term clinical disease progression in HIV-1-positive patients taking combination antiretroviral therapy: the EuroSIDA risk-score. Aids 21: 1867–1875. 17721094 10.1097/QAD.0b013e328270b877

[25] Loutfy MR, Genebat M, Moore D, Raboud J, Chan K, et al (2011) A CD4+ cell count <200 cells per cubic millimeter at 2 years after initiation of combination antiretroviral therapy is associated with increased mortality in HIV-infected individuals with viral suppression. J Acquir Immune Defic Syndr 55: 451–459. 10.1097/qai.0b013e3181ec28ff 21105259

[26] Brennan A, Maskew M, MacPhail P, Sanne I, Fox M (2011) Examining the interaction between current CD4 cell count, current viral load suppression, and time on ART and mortality. 18th Conference on Retroviruses and Opportunistic Infections. Boston, 27 Feb-2 Mar (Abstract 555).

